# Abstracts: International Symposium on Immunopathogenesis of Pregnancy, December 6–7, 1997

**DOI:** 10.1155/S1064744997000318

**Published:** 1997


Infectious Diseases in Obstetrics and Gynecology 5:201–207 (1997)
© 1997 Wiley-Liss, Inc.

### International Symposium on Immunopathogenesis of Pregnancy
### December 6–7, 1997
### American Hospital of Paris
### 63, boulevard Victor-Hugo
### 92200 Neuilly-sur-Seine, France

### Abstracts

Received 1 October 1997

Accepted 21 October 1997

**Endometriosis: Analysis of 1417 Consecutive Cycles of IVF.**

Spandorfer SD, C Fierro, I Kligman, H-C Liu, A Neuer, SS Witkin, Z Rosenwaks. The Center for Reproductive Medicine and Infertility, Department of Obstetrics and Gynecology, The New York Hospital—Cornell Medical Center, New York, NY.

**Objective:** Endometriosis is a very common cause of infertility and often an indication for in vitro fertilization with embryo transfer (IVF-ET). When patients have severe endometriosis affecting the tubes, infertility is understandable. However, the mechanism of infertility with minimal or mild disease is unclear. Some authors have purported a decline in fecundity after IVF-ET in endometriosis patients in advanced stages of disease.[1] It has also been suggested that treatment of minimal or mild endometriosis in infertility patients who are attempting natural conception is beneficial.[2] The purpose of this study was to evaluate the effect of the presence of endometriosis and the stage of disease on IVF-ET outcome.

**Methods:** 1417 consecutive cycles in 872 patients undergoing IVF-ET at The New York Hospital—Cornell Medical Center from 1989 to 1997 were analyzed in a retrospective study. Patients were identified by the presence of endometriosis as a diagnosis (primary, secondary or the presence of endometriosis) in a computer database. All charts were reviewed for the stage of endometriosis and classified according to ASRM.[3] Patients were treated with standard ovulation induction protocols and underwent IVF-ET according to previously published guidelines. Demographics, stimulation protocols and results, retrieval and transfer data, and pregnancy outcomes were analyzed. Clinical pregnancy was defined as the presence of a gestational sac. An ongoing pregnancy was defined as a pregnancy continuing beyond the 20th week of gestation. Analysis of variance, Chi square, and student's *t*-test were utilized when appropriate. $P < 0.05$ was considered significant.

**Results:** 1196 (84.4%) of the 1417 initiated cycles went to retrieval. 1105/1196 (92.3%) underwent an embryo transfer. An overall clinical pregnancy rate/transfer of 44.7% (495/1105) and an overall ongoing pregnancy rate/transfer of 37.1% were noted. In analyzing all cycles that went to transfer, the stage of disease had no significant effect on peak estradiol levels, number of mature oocytes or embryos transferred, or pregnancy outcome. As expected, age was found to have a significant effect on IVF-ET outcome. Patients under 40 years old had a significantly higher clinical pregnancy rate/transfer than those 40 and over (48.4% vs. 31.8%; $p < 0.001$). To remove the effect of individual patients with multiple failed cycles and perhaps other unknown contributing causes of infertility, we next analyzed the first cycle of each patient with endometriosis. Once again, the stage of disease had no significant effect on peak estradiol levels, number of mature oocytes or embryos transferred, or pregnancy outcome. In analyzing the population by stimulation protocol, patients with lupreolide acetate down-regulated cycles were found to be younger, stimulate better, and had significantly higher clinical pregnancy rates (48.9% vs. 27.2%; $P < 0.001$). Given that patients with lupreolide acetate down-regulated cycles were found to have better outcomes, we than analyzed these patients to detect if stage of disease had any effect on IVF-ET outcome. In these patients, lower stages of endometriosis were associated with older women that stimulated better, had more oocytes retrieved, and had more embryos fertilized. Despite this, no differences in the ultimate number of embryos transferred or, more importantly, pregnancy outcomes were detected. We then analyzed the 741 cycles of patients with pure endometriosis as the only cause of infertility. In these patients, the stage of disease had no significant effect on peak estradiol levels, number of mature oocytes, number of embryos transferred, or pregnancy outcome. We then attempted to analyze for the presence of endometriosis and its affect on outcome after IVF-ET. Given that most of the variability in pregnancy outcomes after IVF-ET is related to age and that until the age of 35 we have implantation rates that are constant, we then analyzed all ICSI cycles (1995–1996) in patients with maternal age 35 years or less and male-only infertility ($n = 460$) and compared this to women 35 years or less with male and endometriosis infertility ($n = 43$, same time period) in an attempt to assess the effect of the presence of endometriosis on outcome. In this separate analysis, the presence of endometriosis did not effect the stimulation, retrieval or transfer parameters. Clinical and ongoing pregnancy rates/transfer were similar (male vs. male and endometriosis 63.5% vs. 56% and 56.7% vs. 46.5%, respectively; $P > 0.05$).

**Conclusion:** We have demonstrated in this large study that the stage of endometriosis may have a slight effect on stimulation (lower stage disease in good prognosis patients had better responses), but, more importantly, no differences in pregnancy outcomes were detected. Furthermore, the mere presence of endometriosis as a factor of infertility did not appear to affect IVF-ET outcomes.

1. Fertil Steril 65:603
2. N Engl J Med 337:217
3. Fertil Steril 67:817, 1997.

**Decidualization Related Expression of Plasminogen Activators in Human Endometrial Stromal Cells.**

Papp CS, Toth-Pal E, Schatz F, Lockwood C, Papp Z. 1st Dept. of Ob/Gyn, Semmelweis Medical School, Budapest, Hungary (C.S.P., E.T.-P. Z.P.); Dept. of Ob/Gyn, New York University Medical Center, New York (F.S., C. L.).

To determine whether altered expression of the tissue type (tPA) and urokinase type (uPA) plasminogen activators accompanies decidualization, confluent monolayers of endometrial stromal cells from predecidualized human endometria were incubated for 3 days in a defined medium containing $10^{-8}$M E2, $10^{-7}$M medroxyprogesterone-acetate (MPA), E2+MPA, or 0.1% ethanol as control. It was detected by specific ELISAs that added MPA reduced uPA levels by 19% and tPA by 33%. Even greater reductions were seen in response to E2+MPA (74% for uPA and 49% for tPA). A chromogenic assay was used to discriminate between the amounts of fibrin-independent PA activity (uPA) and fibrin-dependent activity (tPA) in the stromal cell conditioned medium. Two hours preincubation at 37°C increased measured uPA activity under control conditions by about 20-fold, indicating that the stromal cells released uPA predominantly as catalytically inactive pro-uPA. Both uPA and tPA activity were refractory to E2, inhibited by MPA, and synergistically inhibited by E2+MPA. Interestingly enough, E2+MPA reduced the activities of secreted uPA and tPA to nondetectable levels despite significant immunogenic levels of both PAs. This preferential inhibition of activity versus antigen may reflect the action of the potent PA inhibitor, PAI-1, whose output by the cultured stromal cells is elevated by MPA and even more increased by E2+MPA. During implantation, inhibition of decidual cell expressed tPA, the prime fibrinolytic agent, by the predominant steroids of pregnancy can prevent hemorrhage, whereas reduction in decidual cell derived pro-uPA can lower the paracrine supply to specific trophoblast cell uPA receptors and thereby limit trophoblast invasiveness.

**Monoclonal Antibody Against Phosphatidylserine Interferes with the Differentiation of Choriocarcinoma Models of Trophoblast.** Rote NS, Vogt E, Devere G, And Ng AK. Departments of Microbiology and Immunology and Obstetrics and Gynecology, Wright State University School of Medicine, Dayton, OH, USA.

Many studies have confirmed that both monoclonal and naturally occurring antiphospholipid antibodies (aPLs) react with trophoblast from 1st and 2nd trimester human placenta. Using choriocarcinoma models (BeWo and JAR) that mimic trophoblast differentiation, we investigated the effects of monoclonal aPLs on trophoblast. Differentiating choriocarcinomas externalize phosphatidylserine (PS) that is measurable by the increased binding to the cell surface of annexin V and monoclonal aPLs against PS (aPS). They concurrently externalize endogenous annexin V, which binds to the PS and prevents formation of the prothrombinase complex. Choriocarcinomas also undergo invasion of matrigel-coated filters and intercellular fusion leading to syncytium formation. We have previously shown that aPLs prevent intercellular fusion. We tested the effects of monoclonal aPLs on the invasion of JAR. $2.5 \times 10^4$ JAR cells/0.5 ml was applied to matrigel-coated filters. In some chambers the media was supplemented with monoclonal aPS or control class-matched monoclonals against cardiolipin and bacterial peptidoglycan. Chambers were incubated at 37°C for 24 hours and the filters stained and examined microscopically for cells on the underside. Results were compared to media alone (average: 50 cells/underside of membrane). Negative control monoclonals slightly, but not significantly, affected migration. aPS significantly ($P$<0.001) decreased migration of JAR across the membrane. We concluded that PS is expressed on the trophoblast surface during invasion of extracellular matrix, and aPS inhibits the process. Using immunoperoxidase, we also investigated the effects of aPS on annexin V expression on BeWo. Undifferentiated BeWo expressed cytoplasmic, but not surface, annexin V and did not bind exogenous annexin V. After differentiation, BeWo expressed surface annexin V and bound additional exogenous annexin V. When differentiation occurred in the presence of aPS, there was little detectable endogenous or exogenous annexin V surface binding. Using FITC-conjugated annexin V, aPS removed previously bound annexin V from the trophoblast surface. Control monoclonals against cardiolipin and bacterial peptidoglycan had no effect on annexin V expression. Prothrombin did not bind to undifferentiated cells and only bound slightly to differentiated cells. Preincubation with 3SB increased prothrombin binding. We concluded that PS and annexin V are externalized during trophoblast differentiation, and aPS can remove annexin V, opening prothrombin binding sites. Our data suggest that aPLs against PS can directly affect trophoblast function by limiting the depth of decidual invasion and concurrently creating a procoagulant surface on trophoblast exposed to the maternal circulation.

**Production of Signal Transducers Differs Between Cultured Cervical and Myometrial Cells Incubated With Prostaglandin E2.** Lejeune V, Dallot E, Ferrè F, Cabrol D. INSERM U361, Pavillon Baudelocque, Maternité Port Royal-Cochin, Université René Descartes, Paris, France.

Prostaglandin E2 (PGE2) plays a key role in parturition, and administration of a PGE2 agonist at any point of pregnancy can induce delivery. PGE2 receptors are pharmacologically divided into at least four subtypes, EP1, EP2, EP3 and EP4, on the basis of the responses to various agonists and antagonists, and all types have been described to be coupled with G proteins. These subtypes differ in signal transduction, and they are presumed to be coupled with $Ca^{++}$ mobilization (some via inositol triphosphate [IP3] synthesis) and stimulation or inhibition of adenyl cyclase. Therefore, the pharmacological effects of PGE2 are quite diverse. PGE2 can induce muscular contraction via IP3 production and Ca++ mobilization, or it can induce muscular relaxation and cervical maturation via cyclic AMP (cAMP) production. In this study, we examined the production of cAMP and inositol phosphates (IPs) by cultured myometrial and cervical cells, incubated with increasing concentrations of PGE2. Myometrial and cervical cells maintained in culture from uterine biopsies have already been described by our laboratory to maintain a high degree of differentiation, only if cultured within the last 3 days in a serum free-medium. There is a dose-dependent increase in cAMP production by cervical cells, much higher than by myometrial cells. In contrast, PGE2 induces a dose-dependent increase in IP production by myometrial cells, much higher than in cervical cells. When myometrial cells are maintained in the presence of serum, they lose their muscular phenotype, and their response patterns become like those of cervical cells. These results suggest that PGE2 utilizes different signal transduction pathways in myometrial and cervical cells, which have been cultured in good differentiation conditions. Therefore, the actions of PGE2 lead to active muscular contraction in myometrial cells and relaxation in cervical cells, and both of these contradicting actions are necessary for parturition.

**Free Fatty Acids and Alpha-Fetoprotein Profiles in the Human Maternal-Fetal-Placenta Unit at Term Pregnancy.** Benassayag C, Rigoud V, Civel C, Mignot TM, Haourigul M, Hassid J, Leroy MJ, Nunez EA, Ferre F. INSERM U361, Maternité Baudelocque, Universite René Descartes, 75014 Paris, France (C.B., V.R., C.C., T.M.M., M. H., M.J.L., F.F.); Laboratoire de biochimie endocrienne, Facilté de Médecine Xavier Bichat, Université Denis Diderot, 75870 Paris, France (J.H., E.A.N.).

Free fatty acids (FFA), especially polyunsaturated fatty acids (PUFA), play a major role in multifactorial processes involved in normal gestation, fetal development, and parturition. PUFA are cell signaling molecules which are involved in the mediation of immunological, metabolic, and endocrine signals within the materno-feto-placental unit. They act as immunomodulators in the local regulation of maternal cell-mediated immunity, and as growth factors influencing the proliferation of smooth muscle cells. PUFA are precursors of eicosanoids. They also modulate the activity of key enzymes of steroid metabolism and the way in which these hormones act in the myometrium. Consequently, PUFA are directly or indirectly involved in the control of myometrial activity at the time of parturition. PUFA, like arachidonic (C20:4w-6) and docosahexaenoic acids (C22:6w-3), bind with high affinity to the alpha-fetoprotein (AFP) in fetal circulation. AFP, as PUFA, is also among the substances claimed to influence cell growth, to participate in inflammatory reactions, and to act as regulators of the immune response. The distribution of FFA and AFP were studied in each compartment of the maternal-fetal unit on women (n = 28) who underwent elective cesarean section at term before the onset of labor. The study focused on the fetal-maternal interface, the maternal intervillous blood (I), space delineated both by the fetal syncytiotrophoblast and by maternal decidua. The peripheral maternal (M) and fetal circulations (umbilical arteries [A] and vein [V]) were comparatively analyzed. The results indicate a differential distribution of FFA and AFP. The plasma FFA levels were similar in I (725±62μM) and M (760±70μM), but those of A (250±30μM) and V (225±20μM) were significantly lower ($P$<0.001). Saturated FFA were higher in V (41%) and A (44%) than in M (30%) and I (32%). Monounsaturated FFA were predominant in M (43%) and lower in

I (35%), V (33%) and A (31%). Di- and tri-unsaturated FFA were the same in I (25%) and M (25%) and lower in V and A (16%). The most striking differences concerned polyunsaturated FFA, which were low in M (2.5%) and three-fold higher in I, A and V (10%, $P<0.001$); consequently the concentration of C20:4 and C22:6 were three-to-four-fold higher in intervillous space (40 and 16μM) than in M (12 and 4μM) or in cord blood (13μM and 5μM). Immunoquantification of AFP showed a transplacental gradient. The AFP concentrations is high in V and A and also, surprisingly, in the intervillous blood. Very low concentration of AFP were detected in M. Thus the presence of the fetal protein which strongly bind PUFA is correlated with the high percentage of this class of FFA. Hence, a special AFP conformation (less immunoreactive and more anionic) is evidenced in the intervillous space, suggesting that AFP is heavily loaded with PUFA at the fetal-maternal interface. These results suggest that the fetal-maternal interface, with its high levels of AFP and PUFA, could play a key role in the maternal-fetal immune interaction; these factors would enable the fetal allograft to survive or trigger parturition.

**Investigation of *Chlamydia trachomatis* Specific Antibodies in Mothers with Stillbirth.** Gencay M, Koskiniemi M, Puolakkainen M, Ammala P, Wahlstrom T, Vaheri A, Narvanen A. Haartman Institute, Department of Virology and Pathology (M.G., M.K., M.P., T.W., A.V.); University Central Hospital, Obstetrics and Gynecology (P.A.); University of Helsinki and Labsystems Research (A.N.), Helsinki, Finland.

**Objective:** Stillbirth often remains without identified cause. The aim of this study was to find out the association between stillbirth and maternal serological status.
**Methods:** A total of 90 consecutive mothers with stillbirth between July 1994 and March 1997 were prospectively studied. Serum samples of 77 mothers were studied for *C. trachomatis* antibody using a peptide based enzyme immunoassay (EIA), and 73 of them with both EIA and Western blotting. In the peptide based EIA, 12 amino acids long synthetic peptides from the VP4 region of *C. trachomatis* MOMP were used as antigen in solid phase indirect EIA. The serum samples were also tested for human parvovirus B19, HSV-1, HSV2, VZV, cytomegalovirus and *Toxoplasma gondii* antibody using EIA, and for *C. pneumoniae* antibody using a microimmunofluorescence test. Maternal medical history was obtained by interview and from hospital records. The fetuses were autopsied.
**Results:** We found IgG antibodies to *C. trachomatis* in 49% of mothers with stillbirth compared to 23% in fertile age controls ($P<0.01$). IgG antibodies were detected in 47% and IgA in 25% of cases compared to 21% in controls ($P<0.01$ and $P<0.05$) respectively. Sensitivity of the peptide EIA test was 90% and specificity 77% when reactivity with *C. trachomatis* MOMP in immunoblot was used as a reference. The *C. trachomatis* seropositive stillbirth group presented more often with maternal history of *C. trachomatis* infection than the seronegative stillbirth group (13% vs. 5%). Seroprevalence of other antibodies was similar in the two groups, except IgG antibodies to *T. gondii* which were more frequent in the *C. trachomatis* seropositive mothers than in the seronegatives ($P<0.05$).
**Conclusion:** *C. trachomatis* seropositivity was significantly associated with stillbirth. Maternal history of laboratory-confirmed genital *C. trachomatis* infection and seropositivity suggest chronic chlamydial infection as an associated agent in stillbirth.

**CMV Serologic Status of Health Workers.** Vèron M, Antognarelli F, Huraux JM, Fillet AM, Wargon C. Hospital Pitié-Salpetrière, Dept. of Occupational Medicine, Paris, France.

**Objective:** To evaluate cytomegalovirus (CMV) risk of female employees working in health care services (pediatrics unit, nurseries, infectious diseases, kidney transplant unit).
**Methods:** A retrospective analysis of CMV serologic status of women working in exposed services compared to a control group in non-exposed services between 1993–1996 was undertaken at the Department of Occupational Medicine, Hospital Pitié-Salpetrière, Paris, France.. The CMV serologic status was performed at first examination during recruitment or after blood exposure of the agents. 373 women, aged 18–52 years, including nurses, nursing auxiliary staff and physicians were examined.
**Intervention:** To calculate the number of seronegative cases and the seroconversion rate per age classes.
**Main Outcome Measures:** To determine whether or not the contamination was professional or extra-professional. To define the best strategies for primary prevention in women working in exposed services.
**Results and Conclusion:** No seroconversion was observed in the women's cohort. One seroconversion was observed with a man. The percentage of seronegative women aged 18–32 years was 58.5% and 29.3% for women aged 33–52 years. These results emphasize the need for an effective program of primary prevention.

**Three Cases of Second-Trimester Abortion due to B Streptococcus.** Henry-Suchet J, Lokiec-Zerah MH. CHJ. Rostand Sevres (J.H.-C.); Laboratoire Lokiec-Zerah 11 Rue De Cambronne 15e (M.H.L.-Z.).

Invasive B streptococcal infection affects an estimated 3 per 1000 live births. Prenatal morbidity and maternal pathology due to this infection are substantial, but this microorganism is not considered as a cause of late abortion to date. However, some cases have been previously reported by Christensen et al.[1] and Daugaard et al.[2] We report 3 cases of second-trimester infectious abortion, with positive cultures for B streptococcus alone in fetus samples, placenta and amniotic fluid. Of the 3 women, 2 had cervical incompetence and 1 a past history of legal abortions. None of them had urinary infection. In one case, the infection was recurrent, the patient having had 2 previous late abortions with maternofoetal septicemia due to B streptococcus. She achieved a further full term pregnancy after being treated with Augmentin® (amoxicillin-clavulanic acid) during the second trimester of pregnancy. These cases suggest that prevention strategy such as prenatal vaginal screening, maternal immunization or local/general antibiotherapy for B streptococcus carriers should be organized in pregnant women having or susceptible to have a pathological cervix.

1.Christensen et al. (1982)
2.Daugaard et al. (1988).

**Ectopic Pregnancy and Chlamydial Hsp60 Serology—Correlation Between Histopathology, Adhesions and Serologic Responses to 13 Major Epitopes.** Sziller I, Witkin SS, Ziegert M, Csapo Z, Ujhazy A, Papp Z. Department of Obstetrics and Gynecology, Semmelweis University Medical School, Budapest, Hungary; Division of Immunology and Infectious Diseases, Department of Obstetrics and Gynecology, The New York Hospital-Cornell Medical Center, New York, NY.

**Objectives:** To evaluate clinical correlations of immunoreactivity to epitopes of the chlamydial 60 kDa heat shock protein (hsp60) among patients with ectopic pregnancy.

**Method:** In a case-control study, serologic responses to 13 synthetic peptides corresponding to major epitopes of the chlamydial hsp60 were tested in 67 patients treated for ectopic pregnancy.
**Results:** Among patients with ectopic pregnancy, anti-chlamydial serology correlated with histologic evidence of chronic salpingitis (p 0.02), pelvic adhesions (p 0.01), and previous treatment for pelvic inflammatory disease (PID) (p 0.02). Seropositive patients with ectopic pregnancy were more likely than seronegative patients with ectopic gestation to have antibodies to synthetic peptides (p 0.0001). Plasma cell salpingitis was detected in 29 (43.3%) of the ectopic patients. Its presence correlated with antibodies to two hsp60 epitopes, encompassing amino acids 260–271 and 411–422 ($p = 0.02$). Antibodies to these two epitopes, along with 5 other epitopes, also correlated with peritubal adhesion formation in ectopic patients (p 0.01). Antibodies to epitopes 260-271 and 188-199 also correlated with a history of PID ($p = 0.05$). Patients positive for both *C. trachomatis* and hsp60 epitope antibodies had an increased prevalence over controls in their prevalence of salpingitis, pelvic adhesions or history of PID. In contrast, patients who were positive for only *C. trachomatis* antibodies or only hsp60 epitope antibodies did not differ from antibody-negative patients in each of these correlates.
**Conclusions:** Immunoreactivity to specific epitopes within the chlamydial hsp60 correlated with pelvic adhesions, histologic plasma cell salpingitis and a history of PID among women with ectopic pregnancy who were positive for antibodies to *C. trachomatis*. This observation strongly suggests that a *C. trachomatis* infection may be necessary for the induction of hsp60-related tubal immunopathology.

**Cyclic Nucleotide Phosphodiesterases in Human Myometrium: Potential Targets for Relaxant and Anti-Inflammatory Agents.** Leroy MJ, Mehats C, Tanguy G, Robert B, Dallot E, Mignot TM, Duc-Goiran P, Ferrè F. Inserm U361, Pavillon Baudelocque, Université René Descartes, Paris, France.

During human pregnancy, the uterus undergoes considerable structural and functional changes characterized by relaxation along with hypertrophy and hyperplasia of the smooth muscle fibers of the uterine wall. Although the contribution of cyclic nucleotides (cAMP and cGMP) to uterine growth has not been completely determined, increasing evidence suggests that these second messengers play an important role in myometrial relaxation. The phosphodiesterase enzymatic system (PDE) catalyzes and regulates the hydrolysis of cAMP and cGMP and thereby constitutes a critical factor in the control of intracellular concentrations of these messengers. Based on gene families encoding for PDE activity, the superfamily can be divided into at least seven subtypes. Multiple related genes and different mRNA splice forms create various isoforms which are differentially expressed and regulated in individual cell types. From a pharmacological perspective, this complexity implies that individual PDEs are likely good targets for therapeutic intervention in diseases caused or regulated by cyclic nucleotide modulated transduction mechanisms. Cyclic nucleotide PDEs were examined in extracts of human myometrium. Based on the potencies of selective PDE inhibitors, five different PDE families (PDE 1 to 5) have been characterized in the cytosolic fraction of pregnant and non-pregnant tissues. Among them the cAMP-specific PDE family (PDE4) was shown to be the most predominant.[1] Rolipram, which is a selective inhibitor of the PDE 4 family, was also a potent in vitro inhibitor of spontaneous contractions in near term myometrium.[2] To date, four distinct PDE 1 genes (1A, 1B, 1C and 1D) have been identified. Using semi-quantitative RT-PCR, we analyzed the expression pattern of myometrial PDE 4 transcripts in myometrium of pregnant and non-pregnant women. Concurrent expression of PDE 4A, 4B, 4C and 4D mRNAs has been demonstrated. Interestingly, the transcript of PDE 4B is more abundant in myometrium of pregnant than of non-pregnant women, while no difference between both tissues was detected for PDE 4A, 4C and 4D mRNAs. Determining whether the level of expression of PDE 4B mRNA subtype may be directly influenced by steroid hormone modification is under investigation. The physiological implications of these different molecular forms of PDE 4 in the maintenance of pregnancy as well as in the onset of parturition is of particular clinical interest. There are many etiologies for premature birth, but research in this area increasingly points to infectious diseases. A generation of more selective PDE 4 inhibitors with relaxant and anti-inflammatory properties has recently been developed. We can expect that such new drug strategies will be the basis of attempts to improve the treatment of prematurity which still constitutes the major cause of neonatal morbidity and mortality in humans.

1.(Leroy et al, Cell. Signal, 1994, 6, 405).
2.(Leroy et al, Biochem Pharmacol. 1989, 38, 9).

**Expression of Endogenous Retrovirus (ERV-3) mRNA is Coincident with Intercellular Fusion in a Choriocarcinoma Model of Trophoblast Differentiation.** Lin L, Xu B, Rote NS. Departments of Microbiology and Immunology and Obstetrics and Gynecology, Wright State University School of Medicine, Dayton, OH, USA.

ERV-3 is a human endogenous retroviral genome with an open reading frame in the *env* gene that is expressed in placental syncytiotrophoblast and during *in vitro* trophoblast differentiation, suggesting a role in that process. The choriocarcinoma cell line BeWo is a trophoblast model that is undifferentiated and can be induced to undergo differentiation by the addition of forskolin. A 3.5kb ERV-3 *env*-specific mRNA is expressed in forskolin-treated cells concurrently with the production of human chorionic gonadotropin (b-hCG) mRNA. In this study, we evaluated the relationship between differentiation-related expression of ERV-3 env-specific mRNA and intercellular fusion. Cell fusion was determined by immunofluorescent staining for the loss of intercellular membranes by antibodies against E-cadherin; by 48 hours with forskolin, 80% of cells were fused to form syncytia. A digoxigenin-labeled ERV-3 cRNA probe was used in *in situ* hybridization to study the localization of ERV-3 mRNA in BeWo cells undergoing fusion. ERV-3 mRNA was expressed in all cells undergoing intercellular fusion and was not expressed in undifferentiated BeWo cells. Expression of ERV-3 mRNA increased progressively and in parallel with increasing syncytium formation and was maximum at 72 hours. *In situ* hybridization controls included sense and antisense probes and treatment of BeWo cells with RNase. The localization of ERV-3 env-specific mRNA is related to intercellular fusion, suggesting that ERV-3 could play a role in that process. To study further this relationship, we established BeWo sublines containing a plasmid with either sense or anti-sense ERV-3 *env*-specific mRNA. The effects of overproduction of ERV-3 *env*-specific mRNA are currently under investigation.

**Maternal Serum Early Pregnancy Factor in Multiple Pregnancies.** Zheng Zhen-Qun, Fan Xiao-Guang, Ma Ai-Ying, Qiao Chang-Xin. ShanXi Medical University, Talyuan 030001 PR China.

Early pregnancy factor (EPF), a pregnancy associated immunosuppressive molecule, was detected in the pregnant serum of a mouse within 6 hours of mating, and its activity was also detected in the

TABLE 1. Mean value of EPF

| Period of pregnancy | Twin pregnancy | Singleton pregnancy |
|---|---|---|
| First trimester | 8.5 (n = 2) | 6.7 (n = 15) |
| Second trimester | 7.3 (n = 4) | 5.4 (n + 15) |
| Third trimester | 5.0 (n = 16) | 4.4 (n = 15) |

Third trimester triple pregnancy EPF; 7.0 (n = 1).

human maternal serum at a very early stage after fertilization. To investigate the normal distribution of EPF in the human body, we have found its activity in pregnant maternal serum, cervical mucus, amniotic fluid and fetal serum. In the present study, we have detected EPF activity in multiple pregnancy maternal serum by rosette inhibition assay (Table 1). EPF activity is measured by its capacity to enhance the ability of anti-lymphocyte antibody ($CD_3$) to inhibit active spontaneous rosette formation between lymphocytes and sheep red blood cells. EPF activity was expressed as rosette inhibition titer (RIT). The results of this study showed that EPF activity in multiple pregnancy is higher than in singleton pregnancy. It is confirmed again that EPF is produced by fetal tissues.

**Immunological Aspects of Viral Infections in Pregnancy.** Zaidieva ZS, Dantchenko OV, Tioutiohnnik VL, Ignatchenko AA, Krochinina NL. Research Centre of Obstetrics, Gynecology and Perinatology, Moscow, Russia.

The need to study genital herpes virus (HSV) and cytomegalovirus (CMV) is mandated by a continuing increase of the number of patients affected by these disorders, the high rate of perinatal losses and the birth of babies with severe brain damage. We have studied 182 pregnant women with HSV and CMV; 167 patients had initial manifestation of infection in the course of pregnancy. Herpes infection which occurred after the 32nd week of pregnancy has affected 20% of the newborns, while infection occurring shortly before labor has infected 40% of the newborns. All patients have been diagnosed using immunological methods. Analysis of the subpopulations of peripheral blood lymphocytes in pregnant women with genital herpes revealed a reduction in the levels of T-lymphocytes (CD3+), an increase in the levels of T-suppressor cells (CD8+) and B-cells (CD19+) and a significant reduction in the level of T-helper cells (CD4+). No significant deviations from normal levels of these cells were detected in women with reactivation of CMV infection. The choice of treatment methods and optimum ways of delivery are currently of the major interest. We have developed a unique method of immunoglobulinotherapy. We have used this method to treat 170 patients with a history of pregnancy loss caused by generalized infection, persistent spontaneous abortions and non-developing pregnancy. In particular, we have prescribed intravenous injections of immunoglobulin (3 gm every other day for 7 days). The comparison of the state of the immune system prior to the treatment and after has manifested the tendency to increase the number of T-lymphocytes caused by the increase of the number of T helpers.

**Immunological Studies and Treatment of Women with Unexplained Recurrent Spontaneous Abortions.** Georgieva R, Belchev D, Kinkina S, Minev M. Institute of Biology and Immunology of Reproduction (R.G.); Regional Hospital "Dr. R. Angelov" (D.B., S. K.); "Pirogov" Emergency Medical Institute (M.M.), Sofia, Bulgaria.

Recurrent spontaneous abortions (RSA) are a complicated, multifactorial and unsolved problem. An immunologic etiology for this form of abortion has been proposed. In this study, after screening of infertile couples with RSA, 65 couples were classified in the first group: those couples in which a woman had no child born by her partner, the abortions occurred prior to the 20th week of gestation and the women had no lymphocytotoxic antibodies in their serum. The average age of these couples was 26.2 years for women and 31.0 for the men. In the second group were 35 women who had at least one pregnancy that resulted in a live birth or stillbirth after 20 weeks of gestation and antipaternal lymphocytotoxic antibodies in the serum. The mean age of this group was 28.5 years for the women and 32.5 for the men. The number of consecutive abortions in both groups varied from 2 to 6. Fertile couples with 2 or more normal deliveries as well as conventionally treated patients were used as controls. The lymphocyte subsets and the kinetics of antipaternal lymphocytotoxic antibodies were followed during the therapy. The couples were HLA typed for A, B, C and DR antigens. The treatment of the first group consisted of leukocyte immunization including intravenous application of $3X10^8$–$3X10^9$ husband's or donor's WBC (in volume of 50–200 ml) 1–2 times prior to conception and intravenous infusions 2 to 4 times during pregnancy in 4–6-week intervals. The women in the second group were treated with low doses of heparin, 2500 U to 5000 U s.c. twice daily at 12-hour intervals, from the beginning of pregnancy up to 34th or 35th week of gestation. Successful pregnancy was obtained in 81.5% of the immunized women and 75% of the heparin-treated women. All babies are normal and alive up to today. Maternal-paternal HLA-antigen sharing at 2 loci was found to be significantly different from that in normal pregnancy. After immunization, the sera from 28% of women were seroconverted to antipaternal lymphocytotoxic antibodies. The CD8+ lymphocytes were decreased in women with RSA and CD4+/CD8+ ratio increased. A slight reduction in the content of CD11b+ and IILA-DR+ cells, expressing activation markers was found in some patients. There were no significant changes depending on the therapy.

**Blockage of Implantation in the Rat by a Monoclonal Antibody (MAG).** Nemeth G, Kliman HJ, Naftolin F. Department of Obstetrics and Gynecology, Albert Szent-Gyorgyi Medical University, Szeged, Hungary (G.N.); Department of Obstetrics and Gynecology, Yale University, School of Medicine, New Haven, CT, USA (H.J.K., F.N.).

**Introduction:** Implantation is the process by which the blastocyst becomes intimately attached to the endometrium. Synchronization between embryonic and uterine development ("implantation window" or "receptive phase") is necessary for successful implantation in the rodent and human. Recently our group discovered that normal cycling endometrial glands contain an epitope found in the Golgi apparatus of the cell that is strongly reactive with IgG antibodies in numerous murine ascites. Since MAG (mouse ascites Golgi) appears on the endometrial surface at the time of the "implantation window," we speculated that it may play a critical role in the initial adherence of the blastocyst to the endometrium at the time of implantation.

**Methods:** Mature female rats were kept on the cycle of 14:10 hour light:dark. Vaginal smears were taken daily between 9:30 and 10 am. No animal was used for an experiment until at least two consecutive four-day cycles had been observed. The anti-implantation effect of MAG was tested by the intraperitoneal injection of 0.4 ml of anti-MAG antibody or anti-MAG-negative ascites fluid administered in late diestrus. The animals were mated on the next day, and on day 15 the animals were sacrificed and the number of implantations was counted.

**Results:** The anti-implantation effect of MAG was almost complete when the anti-MAG was injected in late diestrus, before ovulation and coitus. ($1.8 \pm 1.2$, $n = 5$ vs. $16.8 \pm 0.37$, $n = 5$, $P < 0.01$). The weight per implantation site was almost the same comparing treated and control groups, indicating that the pregnancies proceeded normally if they implanted.

**Conclusions:** Our findings suppose a significant role of MAG in the implantation process, but more studies are needed to elucidate the mechanism of MAG action.

**The Immunology of a Fetus with the Chronic Pyelonephritis of Pregnant Women.** Bystritskaya TS, Melachova TA, Kozayeva TZ. Blagoveshchensk, Russia.

The aim of the work is to determine the immunologic criteria of the intrauterine fetus being infected by pregnant women with the chronic pyelonephritis. 100 pregnant women with chronic pyelonephritis in the third term and their infants (basic groups), 10 healthy women and their infants (control groups) were examined. The quantity of $CD3^+$, $CD4^+$, $CD8^+$, $CD72^+$lymphocytes and immunoglobulins IgA, IgM, IgG in the serum of blood of the pregnant women and from the umbilical cord (arteries and veins were separated) was studied with the help of the method of direct and indirect fluorescence. Under the latent course of pyelonephritis of the pregnant women, the quantity of $CD3^+$, $CD4^+$ $CD8^+$lymphocytes was considerably smaller than in the control group. In the arterial and venous blood from the umbilical cord, the decrease of CD3-lymphocytes happened mainly because of CD8- lymphocytes and $CD72^+$lymphocytes. IgM and IgG were higher than in the control group. The arterio-venous difference was revealed. The decrease of $CD3^+$, $CD4^+$, $CD8^+$ lymphocytes of the pregnant women with the recurrent pyelonephritis was more expressed than with the latent ones or in the control group. In the blood of the infants of these women, the decrease of $CD3^+$, $CD8^+$ and the increase of $CD72^+$ lymphocytes is more significant both in the artery and in the vein. The arteries venous difference is not significant. With the presence of clinical signs of infection of a fetus, the depression of T- and B-system of immunity was established.

**Non-Specific Immunoprophylaxis of the Intrauterine Infection in Pregnant Women With Chronic Pyelonephritis.** Melachova TA, Bystritskaya TS, Sudakov AA. Blagoveshchensk, Russia.

Non-specific immunoprophylaxis of the intrauterine infection (IUI) by means of essential forte was carried out in 30 pregnant women with chronic pyelonephritis in the critical periods of the formation and development of the fetus immune system (the main group). The immunoprophylaxis consisted of 5 courses of treatment. 20 pregnant women in the control group did not take essential forte. The efficiency of the treatment was assessed by the clinical data, the amount of $CD3^+$, $CD4^+$, $CD8^+$, CD16, and $CD72^+$lymphocytes, immunoglobulins IgA, IgM, and IgG and placental proteins PAMG-1 and AMGF in the blood of the pregnant women and infants. The increase of the absolute amount of $CD3^+$, $CD4^+$, $CD8^+$lymphocytes and the decrease of the absolute amount of $CD72^+$lymphocytes and the placental proteins were revealed in the course of treatment in the main group. In the blood of the infants of these mothers we revealed considerable differences in the amount of immunocompetent cells and the placental proteins in comparison with the infants of the control group. The number of the infants with IUI in the main group was 1.5 times less than in the control group.

**The Immunological Criteria of Preclinical Forms of Gestoses in Adolescents Primiparous.** Kozayeva TZ, Bystritskaya TS, Pozhvdayev VV. Blagoveshchensk, Russia.

The purpose of this investigation is to study the state of cellular and humoral factors of immunity in adolescents primiparous (AP) with the gestosis for early diagnostics and prophylaxis. 40 AP were examined during their pregnancies. In 30, a gestosis of an easy form developed (the basic group). In 10, pregnancy was taking its normal course (the control group). For the determination of CD3, CD4, CD8, CD72 lymphocytes in the serum of blood, the reaction of direct and indirect immunofluorescence with monoclonal antibodies was used. The content of immunoglobulin (Ig) in the blood was studied by the method of radial diffusion in gel (method of Manchini). In AP in the first term of pregnancy, the absolute quantity of CD3, CD4, and $CD72^+$lymphocytes was higher than in the pregnant women of reproductive age of the Far-Eastern region.[1] The increase of the quantity of $CD3^+$lymphocytes in AP was due to $CD8^+$lymphocytes. The content of Ig A, IgM and IgG was not considerably different. In the AP of the basic group during the 21st week of pregnancy, the absolute quantity of CD3 lymphocytes increased in comparison with the quantity of these cells in the control group (P 0.05). The content of IgG and IgM increases two times. It is the diagnostic test of the preclinical forms of gestosis. In the third term of pregnancy in AP, the absolute quantity of $CD3^+$lymphocytes did not truly differ from their quantity in the first term, but the change of ratio of $CD4^+$ and $CD8^+$lymphocytes due to the decrease of $CD8^+$ was revealed. The content of IgG increased two times. With the appearance of clinical signs of gestosis, changes in T-and B-systems of immunity are more expressed.

1.(T.S. Bystritskaya 1993).
